# Multifocal splenic lesions in primary AL amyloidosis secondary to amyloidosis with giant cell granulomatous reaction

**DOI:** 10.1002/jha2.147

**Published:** 2020-12-07

**Authors:** Mishi Bhushan, Kirthi R. Kumar

**Affiliations:** ^1^ Hematopathology Medical City Dallas Medical City Children's Hospital Dallas Texas; ^2^ Hematopathology, Forward Pathology Solutions Medical City Dallas Medical City Children's Hospital Dallas Texas

**FIGURE 1:**
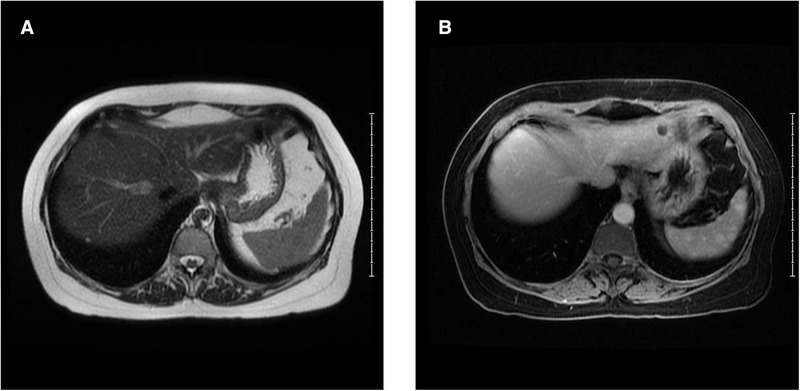
MRI of the abdomen T2 images showed splenic lesions as subtle hypo‐intensity (A). Focal splenic lesions became conspicuous on delayed post‐contrast images with enhancement (B) compared to the background parenchyma

**FIGURE 2:**
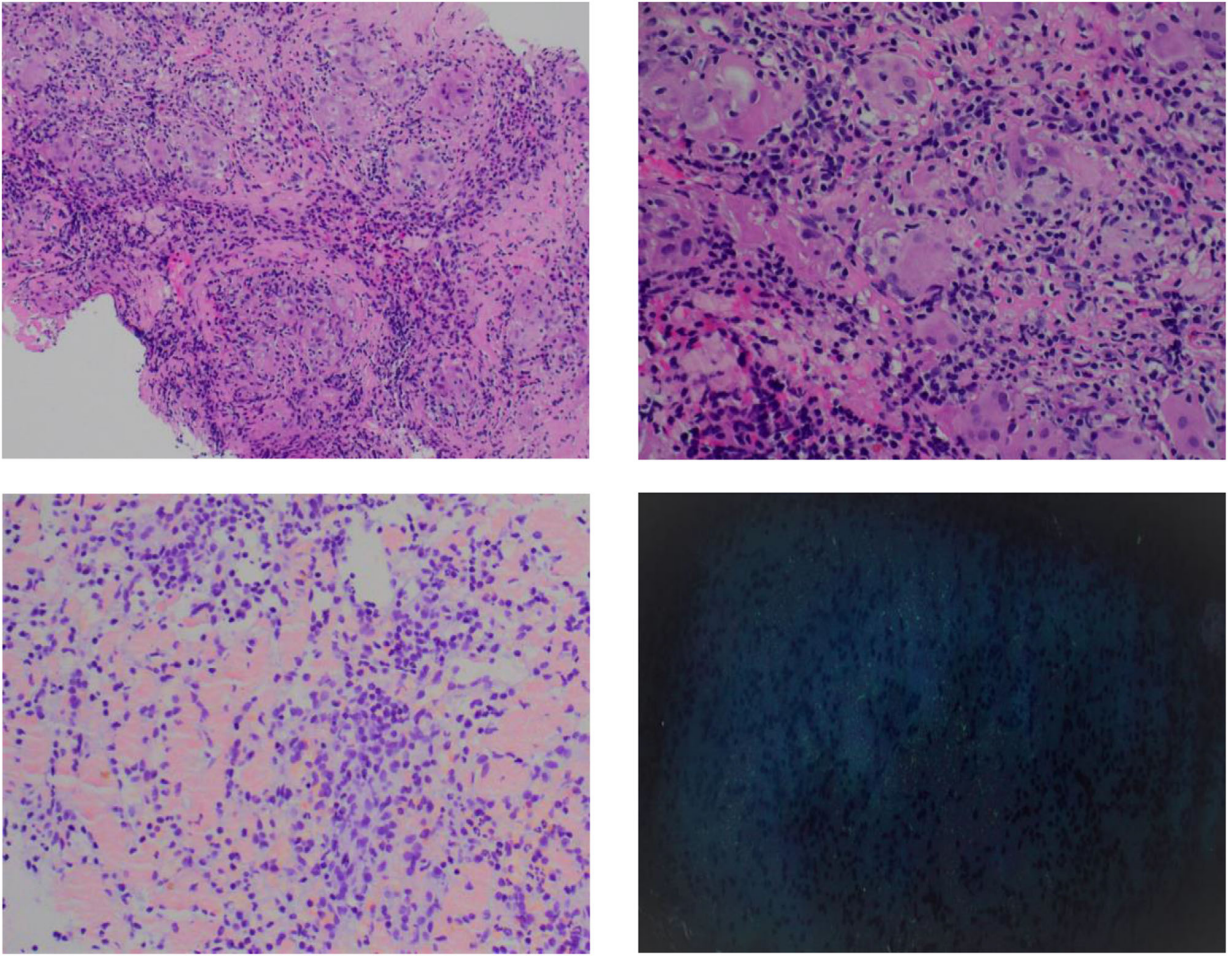
Top left‐low power, top right‐high power, bottom left Congo red, bottom right polarized with apple green birefringence

A 63‐year‐old female with a remote history of clinically inactive lupus was evaluated for monoclonal gammopathy with nephrotic range proteinuria. Bone marrow exam showed 15% lambda restricted plasma cells with normal karyotype and monosomy 13 on fluorescent in‐situ hybiridization analysis. Renal biopsy showed glomeruli with segmental mesangial expansion by amorphous, acellular, and eosinophilic material, which was Congo red positive, without crescents, tubular atrophy, interstitial fibrosis, or inflammation. Immunofluorescence showed segmental staining in the mesangial regions and in the walls of arteries and arterioles for lambda light chain (4+), confirming amyloid light chains (AL) amyloidosis. Positive emission tomography scan performed to evaluate for skeletal disease showed multiple hypermetabolic focal lesions in the spleen with a maximum standard uptake value of 5.5 units. Magnetic resonance imaging (MRI) confirmed greater than 15 splenic lesions that were slightly hypointense on T2 imaging and were isointense on non‐contrast T1 imaging. Lesions were not apparent on the early phase contrast images (Figure 1A) and became conspicuously hyperintense on delayed imaging (Figure 1B). Needle core biopsy of splenic lesion demonstrated multiple scattered small noncaseating giant cell granulomas composed of foreign body giant cells, histiocytes, surrounded by chronic inflammatory cell infiltrate (Figure 2). Congo red showed diffuse congophilic deposits with apple‐green birefringence on polarization; some foci showed co‐localization with the giant cells. Special stains for acid fast and fungal elements (acid‐fast bacilli Fite and Gomori Methenamine Silver) were negative. Immunohistochemistry with CD3 and CD5, CD20, and PAX5, and CD138 showed moderate numbers of T, B, and plasma cells, respectively. Interestingly, an atypical plasma cell infiltrate was not identified. Flow cytometry did not show any abnormal B‐ or T‐ cell populations. Mass spectrometry on peptides extracted from Congo red positive areas of paraffin‐embedded splenic tissue detected presence of a peptide profile consistent with AL lambda type amyloid deposition.

Primary AL amyloidosis is a multisystemic, heterogeneous disease caused by the deposition of toxic insoluble beta‐sheet fibrillar protein aggregates in various tissues. Incidence of splenic involvement is unknown and splenic amyloidosis usually presents as heterogeneous enhancement and hypoperfusion of the spleen on CT imaging and decreased signal intensity on T2 MRI images. However, this case presented as multifocal lesions that were hypointense on T2 images but showed hyperintense enhancement of lesions on delayed post‐contrast images. Presentation with multifocal enhancing lesions in spleen is rare as is inflammatory reaction with noncaseating granulomas on pathology in patients with amyloidosis. Granulomatous reaction with amyloidosis has been reported previously. The atypical MRI findings in this case were likely related to the granulomatous inflammation secondary to amyloid deposition. This is an unusual presentation of splenic findings in a patient with primary amyloidosis.

